# Treatments of Squamous Cell Cancer

**DOI:** 10.3390/cancers12113229

**Published:** 2020-11-02

**Authors:** Darido Charbel

**Affiliations:** 1 The Peter MacCallum Cancer Centre, 305 Grattan St, Melbourne 3000, VIC, Australia; Charbel.Darido@petermac.org; 2 Sir Peter MacCallum Department of Oncology, The University of Melbourne, Parkville 3010, VIC, Australia

It is now clear that the most common solid cancer is squamous cell cancer (SCC) [1]. This malignant tumour originates mainly from epithelial cells that cover the skin, the surfaces of the respiratory and digestive tracts, and the linings of the hollow organs of the body that interface with the external environment. These epithelia are constantly challenged within diverse anatomical locations and share a common genetic mutational landscape [2]. Among the etiological factors of SCC, UV irradiation, exposure to carcinogen such as tobacco smoking and betel quid chewing, frequent alcohol use, genetic predisposition, immunosuppression, and the diverse commensal microbiome are highly cited. Treatments of SCC that consider the disease molecular drivers and its microenvironment have been embraced to overcome SCC heterogeneity [3].

Current conventional treatment regimens for SCC, including surgery, radiation, chemotherapy, targeted therapy and immunotherapy, are non-selective and are administered regardless of biomarkers [4]. Therapy resistance and/or cancer recurrence subsequently emerges leading to SCC patient mortality. Over the last decade, the SCC field has witnessed an unprecedented investment in the development, characterisation and translation of novel biomarkers to have real clinical value [5]. The enduring challenge ahead involves understanding how best to pair these biomarkers with preventative and therapeutic approaches to extract the maximum benefit for SCC patients. Central to this ambition is the knowledge of how to tailor SCC therapies to specific risk factors and molecular drivers while enhancing the immune response to eradicate the disease. 

In this Special Issue, Dr Darido brings together experts in the field of SCC to provide an overview of the current treatment advances. As we develop a better understanding of the limitations of current therapies, we expect to highlight new diagnostic, prognostic/predictive and therapy response/resistance biomarkers for interventional modalities in emerging immuno-oncology therapeutic areas, and areas of unmet clinical need. Future prospects enabling improved therapies will provide an exciting therapeutic roadmap for the control of SCC and will ultimately contribute to alleviating the huge burden of SCC in patients. 
